# Epstein-Barr Virus Promotes Tumor Angiogenesis by Activating STIM1-Dependent Ca^2+^ Signaling in Nasopharyngeal Carcinoma

**DOI:** 10.3390/pathogens10101275

**Published:** 2021-10-03

**Authors:** Jiaxiang Ye, Jiazhang Wei, Yue Luo, Yayan Deng, Ting Que, Xiaojian Zhang, Fei Liu, Jinyan Zhang, Xiaoling Luo

**Affiliations:** 1 Department of Medical Oncology, Guangxi Medical University Cancer Hospital, 71 Hedi Road, Nanning 530021, China; yejiaxiang2013@163.com (J.Y.); ly569770803@126.com (Y.L.); dengmoure@163.com (Y.D.); 2 Department of Otolaryngology & Head and Neck, The People’s Hospital of Guangxi Zhuang Autonomous Region, Guangxi Academy of Medical Sciences, 6 Taoyuan Road, Nanning 530021, China; somnent@gmail.com; 3 Department of Immunology, School of Basic Medical Sciences, Guangxi Medical University, 22 Shuangyong Road, Nanning 530021, China; youxian173@163.com (T.Q.); zxj199509@163.com (X.Z.); 4 Research Center of Medical Sciences, The People’s Hospital of Guangxi Zhuang Autonomous Region, Guangxi Academy of Medical Sciences, 6 Taoyuan Road, Nanning 530021, China; gxfeiliu@hotmail.com; 5 Research Department, Guangxi Medical University Cancer Hospital, 71 Hedi Road, Nanning 530021, China

**Keywords:** Epstein-Barr virus, stromal interaction molecule 1, tumor angiogenesis, nasopharyngeal carcinoma

## Abstract

Epstein-Barr virus (EBV) promotes tumor angiogenesis in nasopharyngeal carcinoma (NPC) by activating store-operated Ca^2+^ entry. Since such entry has been linked to stromal interaction molecule 1 (STIM1), we examined whether the virus acts via STIM1-dependent Ca^2+^ signaling to promote tumor angiogenesis in NPC. STIM1 expression was detected in NPC cell lines HK1 and CNE2 that were negative or positive for EBV. STIM1 was knocked down in EBV-positive cells using recombinant lentivirus, then cytosolic Ca^2+^ levels were measured based on fluorescence resonance energy transfer. Cells were also exposed to epidermal growth factor (EGF), and secretion of vascular endothelial growth factor (VEGF) was measured using an enzyme-linked immunosorbent assay. Endothelial tube formation was quantified in an in vitro angiogenesis assay. Growth of CNE2-EBV xenografts was measured in mice, and angiogenesis was assessed based on immunohistochemical staining against CD31. Paraffin-embedded NPC tissues from patients were assayed for CD31 and STIM1. EGFR and ERK signaling pathways were assessed in NPC cell lines. STIM1 expression was higher in EBV-positive than in EBV-negative NPC cell lines. STIM1 knockdown in EBV-positive NPC cells significantly reduced Ca^2+^ influx and VEGF production after EGF treatment. STIM1 knockdown also inhibited xenograft growth and angiogenesis. Moreover, CD31 expression level was higher in EBV-positive than EBV-negative NPC tissues, and high expression of CD31 co-localized with high expression of STIM1 in EBV-positive tissues from NPC patients. Viral infection of NPC cells led to higher levels of phosphorylated ERK1/2 after EGF treatment, which STIM1 knockdown partially reversed. Our results suggest that EBV promotes EGF-induced ERK1/2 signaling by activating STIM1-dependent Ca^2+^ signaling, and that blocking such signaling may inhibit EBV-promoted angiogenesis in NPC.

## 1. Introduction

Nasopharyngeal carcinoma (NPC) is the most frequent head and neck malignant tumor related to Epstein-Barr Virus (EBV) in southern China, and it is associated with a high risk of local lymph node invasion and distant metastasis [1,2]. While this malignant tendency is often the main reason for the failure of NPC treatment [2,3], the processes driving this malignancy remain to be clarified.

Tumor invasion and metastasis are closely related to tumor angiogenesis [4,5]. Tumor cells secrete a large amount of vascular endothelial growth factor (VEGF) into their microenvironment, which recruits vascular endothelial cells and vascular interstitial cells and thereby leads to abnormal tumor angiogenesis. Since non-tumor cells are also involved in angiogenesis, blocking the interaction between tumor cells and non-tumor cells in the local microenvironment may be a way to block tumor angiogenesis [6,7].

One way that tumor and non-tumor cells communicate is via store-operated Ca^2+^ entry (SOCE) [8,9], and abnormal SOCE-mediated Ca^2+^ signaling is involved in cancer onset and progression [10,11]. Our previous study found that EBV infection can activate epidermal growth factor (EGF)-induced, SOCE-mediated Ca^2+^ signaling and increase VEGF production to promote NPC tumor angiogenesis [12] and blocking SOCE using SKF96365 or 2-aminoethoxydiphenyl borate can inhibit the growth and angiogenesis of NPC [12,13]. Since stromal interaction molecule 1 (STIM1) regulates SOCE channels [14] and influences tumor angiogenesis in cervical and breast cancer [15,16], we wondered whether EBV might act via STIM1-dependent Ca^2+^ signaling to promote tumor angiogenesis in NPC. By exploring this question, the present study may provide new therapeutic strategies against the invasion and metastasis of NPC.

## 2. Materials and Methods

### 2.1. Cell Culture

EBV-positive HK1-EBV and CNE2-EBV NPC cells were kind gifts of Prof. Sai-Wah Tsao (Hong Kong University, Hong Kong, China) and Prof. Musheng Zeng (Sun Yat-sen University Cancer Center, Guangzhou, China), respectively, and had been established as described [17,18]. The efficiency of EBV infection in these EBV-positive cells was validated by testing the expression of EBV-encoded latent membrane protein 1 as described [12]. These two cell lines and the corresponding EBV-negative NPC cell lines HK1 and CNE2 were cultured using Dulbecco’s Modified Eagle Medium (DMEM) supplemented with 10% fetal bovine serum (FBS) at 37 °C under 5% CO_2_. Human umbilical vein endothelial cells (HUVECs; CRL-1730^TM^; American Type Culture Collection [ATCC]; Manassas, VA, USA) were cultured in Endothelial Cell Medium(ECM) (ScienCell, San Diego, CA, USA.) supplemented with 5% FBS. The cell lines in the present study were authenticated through short-tandem repeat profiling [19,20,21].

### 2.2. STIM1 Knockdown

STIM1 expression was assayed in EBV-negative and -positive NPC cells. EBV-positive cells were transfected with recombinant plasmid vector GV248 encoding short hairpin RNA against STIM1 (shRNA-STIM1) or negative control short hairpin RNA (shRNA-NC). Lentivirus transfections were carried out according to the instructions provided by the manufacturer (GeneChem Technology, Shanghai, China). STIM1 expression was assayed by Western blotting with an anti-STIM1 antibody (1 mg/mL, cat. no. ab57834, Abcam, Cambridge, UK). β-actin was assayed as an internal control using an antibody (1 mg/mL, cat. no. ab8226, Abcam).

### 2.3. Cytosolic Ca^2+^ Measurement

Cytosolic Ca^2+^ levels were measured in serum-deprived EBV-positive NPC cells stably transfected with shRNA-NC or shRNA-STIM1 and exposed to 50 ng/mL recombinant human EGF (PeproTech, Cranbury, NJ, USA). Levels were monitored using the Ca^2+^ probe YC 3.60 and fluorescence resonance energy transfer (FRET) [22] as described in our previous study [23]. The YC3.60 Ca^2+^ sensor was activated by an argon laser operating at 440 nm, and emission was measured at 480 nm for cyan fluorescent protein (CFP) as well as at 530 nm for FRET-dependent Venus. FRET measurements were taken using an AquaCosmos imaging and analysis system (Hamamatsu Photonics, Hamamatsu, Japan). The YC 3.60 probe is more suitable for long-term observation of Ca^2+^ levels than the traditional fluorescent indicator Fura-2/AM [23].

### 2.4. Assays of VEGF and Tubule Formation

EBV-positive NPC cells stably transfected with shRNA-NC or shRNA-STIM1 were cultured in a six-well plate (5 × 10^4^ cells per well). When cells had grown to 85% confluence, they were pre-incubated for 24 h in serum-free medium, then for 12 h in DMEM containing 50 ng/mL EGF or 0.25% FBS in order to induce VEGF production. The amount of VEGF in the medium was assayed using the VEGF Human ELISA Kit (FANKEL Industrial, Shanghai, China) according to the manufacturer’s instructions. The medium was also assayed for tubule formation as described [12]. The total number of tubes per standard field in each well was calculated using Image J analysis software (developed by Wayne Rasband, National Institutes of Health, Bethesda, MD, USA).

### 2.5. Xenograft Model in Mice

All animal experiments were approved by the Animal Research Committee of Guangxi Medical University and were carried out according to the regulations of the Animal Research Committee of Guangxi Medical University. CNE2-EBV cells stably transfected with shRNA-STIM1 or shRNA-NC were subcutaneously inoculated into, respectively, the right and left dorsal sides of female BALB/c (nu/nu) mice 4–6 weeks old (5 × 10^6^ cells per side). Xenograft size was measured every 5 days from the macroscopic tumor, and xenograft volume was calculated according to the following formula: *V* = (*W*^2^ × *L*)/2, where *W* indicated the width and *L* the length. The mice were sacrificed and their tumors were isolated at the end of the observation. Tumor sections were immunostained for the vascular endothelial marker CD31 using an anti-CD31 antibody (1:5000, cat. no. ab281583, Abcam).

### 2.6. Clinical NPC Tissues

After the study was approved by the Ethics Committee of Guangxi Medical University Cancer Hospital and patients provided informed consent, samples from 29 patients diagnosed with undifferentiated NPC (15 EBV-positive, 14 EBV-negative) were collected before treatment. Among the patients, 15 were diagnosed with EBV-positive disease based on positivity for EBV viral capsid antigen (VCA)-IgA and EBV-DNA ≥ 1000 copies/mL, while 14 were diagnosed with EBV-negative disease based on lack of detection of EBV VCA-IgA and EBV-DNA < 1000 copies/mL. Expression of CD31 was detected by immunohistochemical staining using an anti-CD31 antibody (1:100, cat. no. M0823, Agilent Dako, Santa Clara, CA, USA). The average number of CD31-positive microvessels in all visual fields was calculated as described [24].

The relationship between STIM1 expression and tumor angiogenesis was explored by immunostaining EBV-positive NPC tissue against STIM1 and CD31 with anti-STIM1 antibody (1:100, cat. no. 11565-1-AP, Proteintech, Wuhan, China) and anti-CD31 antibody (1:100, cat. no. M0823, Agilent Dako), followed respectively by the secondary antibodies Alexa Fluor^®^ 488-conjugated goat Anti-Rabbit IgG (H+L) (1:400, cat. no. GB25303, Servicebio, Wuhan, China) or Cy3-conjugated goat anti-mouse IgG (H+L) (1:300, cat. no. GB21301, Servicebio). Immunofluorescence images were captured using the TissueFAXS Plus Imaging system (TissueGnostics, Vienna, Austria).

### 2.7. Measurement of Ca^2+^-Related Signaling Pathways in NPC Cells

The mRNA expression profiles of head and neck cancer (HNSC) tissues were downloaded from The Cancer Genome Atlas (TCGA) database via UCSC Xena (https://xenabrowser.net/datapa-ges/; accessed on 20 November 2020), and gene pathways most closely related to STIM1 were identified using gene set enrichment analysis (GSEA). HNSC cases were divided into those expressing low or high STIM1. Moreover, genes differentially expressed (DEGs) between EBV-negative or -positive NPC cells were analyzed using transcriptome sequencing data downloaded from the NCBI SRA database (accession: SRX3199730). DEGs were defined as those showing a | log_2_(fold change)| > 2 based on the fragments per kilobase of exon per million fragments mapped (FPKM). Pathway enrichment of upregulated DEGs was analyzed using the Kyoto Encyclopedia of Genes and Genomes (KEGG) and the David online tool (https://david.ncifcrf.gov/; accessed on 20 January 2021).

The EGF receptor (EGFR) signaling pathway was assayed based on levels of EGFR and phosphorylated EGFR (p-EGFR) in HK1 and CNE2 NPC cells and in the corresponding EBV-positive cell lines, which were stably transfected (or not) with shRNA-NC or shRNA-STIM1 and which were stimulated (or not) with EGF. The ERK1/2 signaling pathway was assayed based on levels of ERK1/2 and p-ERK1/2 in the same NPC cell lines stimulated with EGF. Western blotting was performed using antibodies against EGFR (1:1000, cat. no. 4267, Cell Signaling Technology, Boston, MA, USA), p-EGFR (1:1000, cat. no. 3777, Cell Signaling Technology), ERK1/2 (1:10,000, cat. no. ab184699, Abcam) or p-ERK1/2 (1:400, cat. no. ab223500, Abcam). GAPDH expression was determined as an internal control using an anti-GAPDH antibody (1:10,000, cat. no. ab8245, Abcam).

### 2.8. Statistical Analysis

Data in at least three independent experiments were analyzed using GraphPad Prism 8.1 (GraphPad, San Diego, CA, USA) and SPSS 16.0 software (IBM, Chicago, IL, USA). Continuous data were reported as mean ± standard deviation (mean ± SD). Inter-group differences of data with a Gaussian distribution were assessed for significance using a two-tailed *t*-test unless otherwise stated. Differences associated with *p <* 0.05 were considered statistically significant.

## 3. Results

### 3.1. STIM1 Knockdown Reduces EGF-Induced, SOCE-Mediated Ca^2+^ Influx in EBV-Positive NPC Cells

**Figure 1:**
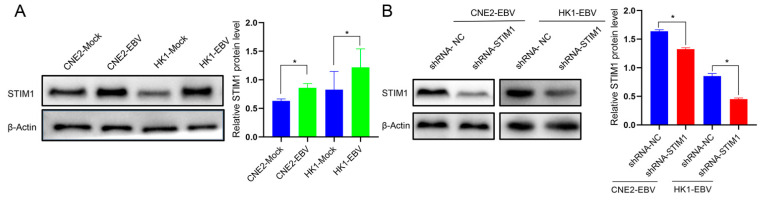

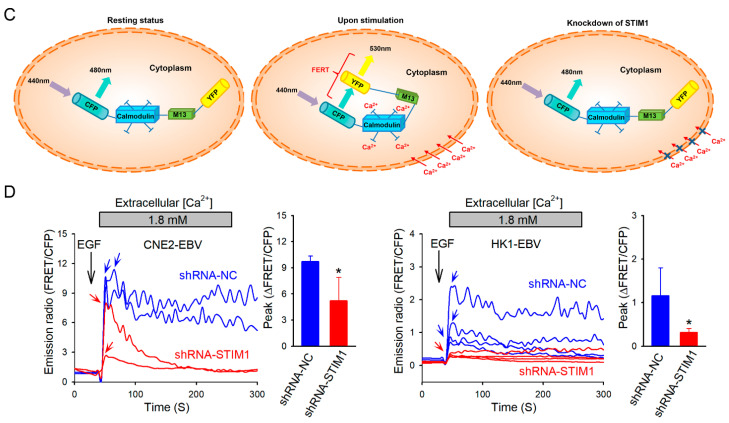
STIM1 knockdown reduces EGF-induced, SOCE-mediated Ca^2+^ influx in EBV-positive NPC cells. (**A**) Levels of STIM1 protein in mock-infected and EBV-infected CNE2 or HK1 cells. Data are shown as mean ± SD (* *p* < 0.05, Student’s *t*-test). (**B**) Effects of STIM1 knockdown in CNE2-EBV and HK1-EBV cells. Data are shown as mean ± SD (* *p* < 0.05, Student’s *t*-test). (**C**) Schematic diagram of FRET signaling in NPC cells expressing YC3.6 probe when cells were at rest, stimulated with EGF, or subjected to SOCE blockade. (**D**) Ca^2+^ levels in EBV-infected NPC cells after STIM1 knockdown were shown as changes in emission of FRET/CFP (ratio of fluorescence emission at 530 nm to 480 nm). The representative Ca^2+^ response curves in the individual cell are shown. The peaks of each curve are denoted with arrows. Intensities were quantified as peak Ca^2+^ levels. Data are shown as mean ± SD (* *p <* 0.05, Student’s *t*-test).

In our previous study, we found that EBV infection of NPC cells facilitated Ca^2+^ influx via SOCE without affecting Ca^2+^ release from intracellular Ca^2+^ stores [12]. Since STIM1 is an essential component of SOCE channels, we first explored the effects of EBV on its expression. STIM1 levels were significantly higher in EBV-positive than EBV-negative NPC cells (Figure 1A). Knocking down STIM1 using a short hairpin RNA (Figure 1B) significantly reduced EGF-induced Ca^2+^ influx via SOCE in EBV-positive cells (Figure 1C,D).

### 3.2. STIM1 Knockdown Decreases EGF-Induced VEGF Production and Endothelial Tube Formation

**Figure 2:**
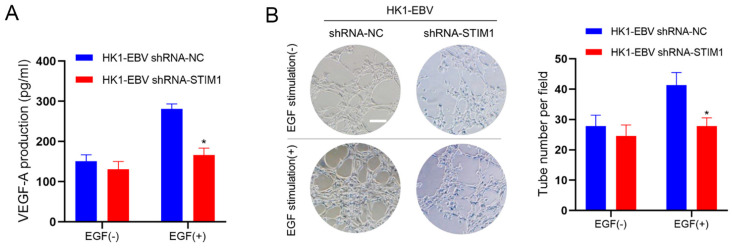

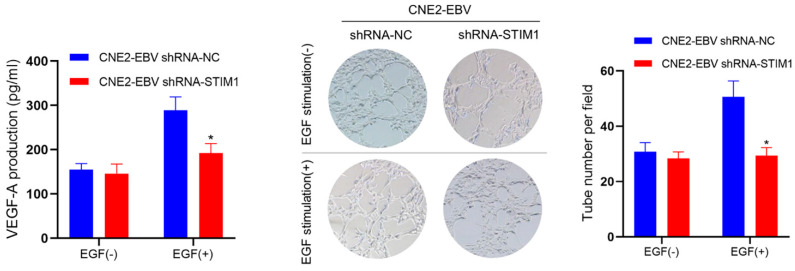
STIM1 knockdown decreases EGF-induced VEGF production and endothelial tube formation. (**A**) VEGF levels in the culture medium of NPC cells stably transfected with negative control short hairpin RNA (shRNA-NC) or short hairpin RNA targeting STIM1 (shRNA-STIM1). Data are shown as mean ± SD (* *p <* 0.05, Student’s *t*-test). (**B**) HUVECs were incubated with the conditioned medium as indicated. Representative photographs of HUVEC tube formation are shown. Scale bar = 100 μm. The number of tubes per standard field was calculated for each well. Data are representative of three independent experiments and are shown as mean ± SD (* *p <* 0.05, Student’s *t*-test).

In our previous study, we found that EBV infection of NPC cells, by amplifying the Ca^2+^ signaling via SOCE, increased EGF-stimulated VEGF production and enhanced endothelial tube formation [12]. In the present study, we found that knocking down STIM1 in EBV-positive NPC cells significantly dampened the ability of EGF to induce VEGF production, without affecting baseline VEGF expression (Figure 2A). STIM1 knockdown also significantly decreased EGF-stimulated endothelial tube formation (Figure 2B).

### 3.3. STIM1 Knockdown Slows Xenograft Growth and Angiogenesis

**Figure 3:**
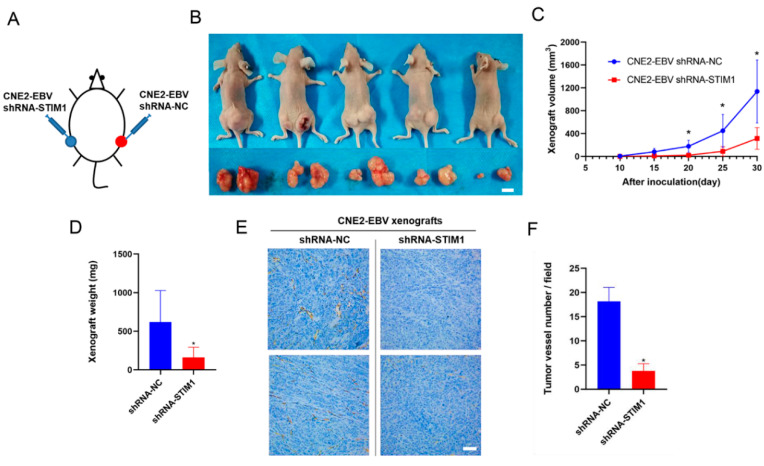
STIM1 knockdown inhibits xenograft growth and CD31 expression. (**A**) Schematic of the shRNA-NC/shRNA-STIM1 xenograft-bearing mouse model. (**B**) Representative photographs of female BALB/c mice harboring both shRNA-NC (right) and shRNA-STIM1 (left) xenografts. Images were captured at 30 days after inoculation. Bottom panels show dissected xenografts. Scale bar = 10 mm. (**C**) Xenograft volumes (*n* = 5) were measured every 5 days from day 10 after inoculation, when xenografts became palpable. (**D**) Xenografts were isolated from sacrificed mice on day 30 and weighed. (**E**) Representative results of immunohistochemistry against CD31 in paraffin-embedded xenograft sections. Magnification, 200×; Scale bar = 100 μm. (**F**) Tumor angiogenesis was quantified in terms of the number of vessels per field. Data are shown as mean ± SD (* *p <* 0.05, Student’s *t*-test).

To examine the effects of STIM1 knockdown in vivo, mice were concurrently inoculated with CNE2-EBV cells at bilateral dorsal sites: shRNA-NC cells were injected on the right side, and shRNA-STIM1 cells on the left (Figure 3A,B). STIM1 knockdown significantly slowed xenograft growth (Figure 3C,D) and reduced CD31 expression (Figure 3E,F).

### 3.4. EBV Infection Promotes Angiogenesis by Upregulating STIM1

**Figure 4:**
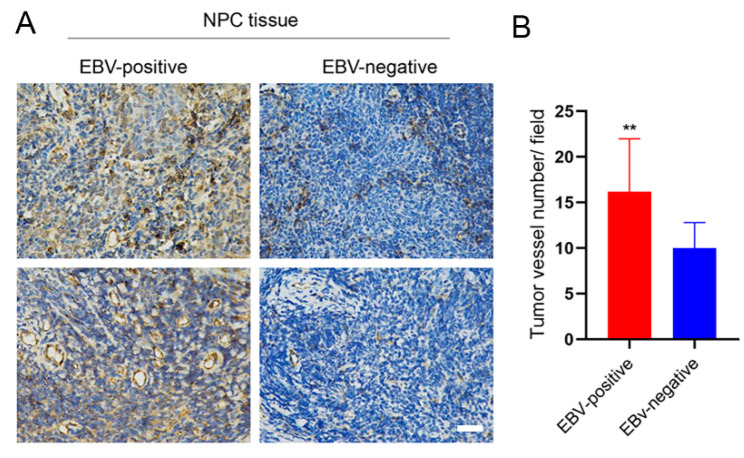
EBV infection promotes the expression of CD31 in NPC tissues from patients. (**A**) Representative results of immunohistochemistry against CD31 in paraffin-embedded NPC tissues from patients. Magnification, 400×, Scale bar = 50 μm. (**B**) Tumor angiogenesis was quantified in terms of the numbers of vessels per field. Data are shown as mean ± SD (** *p <* 0.01, Student’s *t*-test).

**Figure 5:**
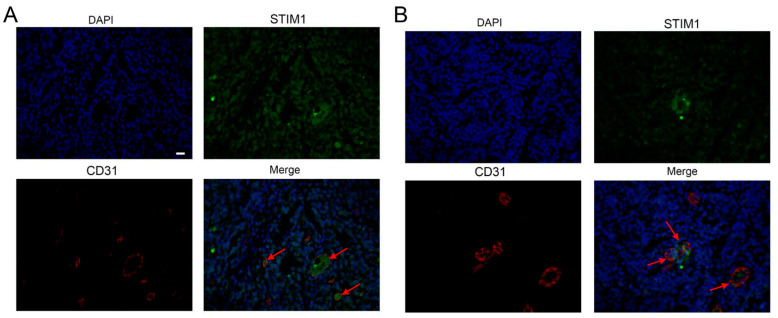
High expression of STIM1 correlates with high expression of CD31. The two panels (**A**,**B**) show immunostained micrographs from paraffin-embedded NPC tissues from two EBV-positive patients. Blue indicates DAPI, green indicates STIM1 and red indicates CD31. Arrows indicate examples of high expression. Magnification, 400×; Scale bar = 50 μm.

To complement our previous studies in vitro and in mice [12], we examined CD31 expression in 15 EBV-positive and 14 EBV-negative NPC clinical samples (Table S1). CD31 levels were significantly higher in EBV-positive than EBV-negative tissues (Figure 4). Moreover, high expression of CD31 co-localized with high expression of STIM1 in EBV-positive tissues from patients (Figure 5). This suggests that greater angiogenesis correlates with higher STIM1 expression in NPC.

### 3.5. EGF Activates the EGFR/p-EGFR Pathway in NPC Cells

**Figure 6:**
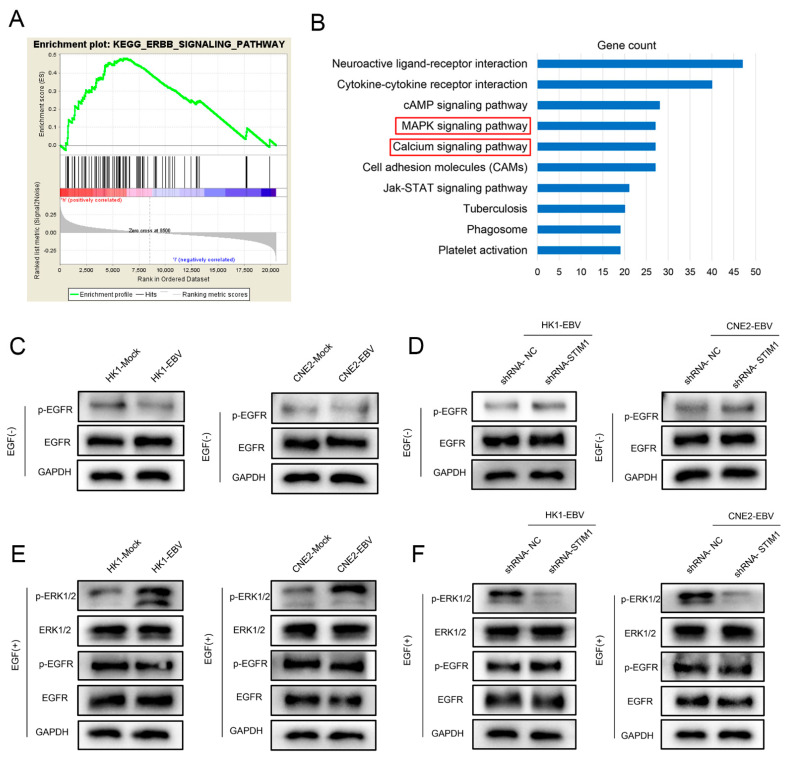
Involvement of EGFR and ERK signaling pathways in NPC. (**A**) GSEA of HNSC transcriptomic data downloaded from TCGA. (**B**) KEGG analysis of genes upregulated in EBV-positive NPC cells relative to EBV-negative cells. (**C**–**F**) Levels of EGFR, p-EGFR, ERK1/2 and p-ERK1/2 in NPC cells. Data are representative of three independent experiments.

The mRNA expression profiles of 522 HNSC cases were downloaded from TCGA, and GSEA showed the ErbB signaling pathway to correlate positively with the gene set in samples showing high STIM1 expression (Figure 6A). In our previous study, we found that EGF activated Ca^2+^ signaling in NPC cells, but the underlying mechanism was unclear [12]. Since EGF activation of the ErbB signaling pathway should promote Ca^2+^ signaling via EGFR (ERB-B1) (Figure S1), we investigated the EGFR/p-EGFR signaling pathway in NPC cells that were stimulated or not by EGF. EGF increased p-EGFR levels (Figure 6C–F). However, p-EGFR levels were unaffected by EBV infection or STIM1 knockdown.

### 3.6. STIM1 Knockdown Partially Reverses EBV-Promoted Activation of the ERK1/2/p-ERK1/2 Pathway

Transcriptome analysis revealed 5412 genes upregulated by EBV infection in NPC cells, and the top 10 KEGG pathways involved Ca^2+^ and MAPK/ERK signaling (Figure 6B). Since the latter is a classic angiogenesis-related signaling pathway [25,26], we measured levels of ERK1/2 and p-ERK1/2 in NPC cells that had been infected (or not) with EBV and in which STIM1 was knocked down (or not). EBV infection increased levels of p-ERK1/2 (Figure 6C,E), whereas STIM1 knockdown reduced them (Figure 6D,F).

## 4. Discussion

Aberrant SOCE-mediated Ca^2+^ signaling can trigger oncogenic signaling pathways that drive tumor proliferation, angiogenesis, invasion and metastasis [27]. Our previous study showed that EBV infection promotes NPC tumor angiogenesis by facilitating SOCE-mediated Ca^2+^ signaling [12]. Confirming those results obtained in vitro and in mice, the present study showed higher CD31 expression in EBV-positive than EBV-negative NPC tissues from patients. We also confirmed here the involvement of SOCE-mediated Ca^2+^ signaling in EBV-driven NPC development by showing that STIM1, an essential component of the SOCE channel, is upregulated by EBV infection in NPC cells and helps mediate the ability of EGF to induce Ca^2+^ influx via SOCE, which in turn drives tumor angiogenesis. STIM1 aggregation is required for SOCE-mediated signaling [28,29]. Our previous study showed that EBV infection enhances STIM1 aggregation in NPC cells [23], and the present study confirms and extends this by showing that infection upregulates STIM1.

VEGF drives tumor angiogenesis [30], and our experiments indicate that STIM1 is important for EBV-promoted angiogenic Ca^2+^ signaling in NPC. Knocking down STIM1 in EBV-positive cells of NPC inhibited EGF-induced VEGF production and endothelial tube formation. We confirmed these results in vivo by showing that STIM1 levels correlated directly with CD31 levels in NPC tissues from patients, and that STIM1 knockdown decreased CD31 expression in xenografts in mice. These effects in mice translated to slower tumor growth.

Transcriptomic analysis of HNSC samples linked STIM1-dependent Ca^2+^ signaling to the EGFR/p-EGFR signal pathway. When we tried to confirm this in vitro, we found that, as expected, EGF led to higher p-EGFR levels, yet neither EBV infection nor STIM1 knockdown appeared to affect EGFR/p-EGFR signaling. This contrasts with the reported ability of the EBV-encoded latent membrane protein to upregulate EGFR in NPC [31,32]. The discrepancy between our results and those in the previous studies may reflect the in vitro nature of our experiments. Therefore, in vivo experiments are needed to examine whether and how EBV acts via EGFR to drive SOCE-mediated Ca^2+^ signaling.

Our transcriptomic analysis suggests that EBV infection drives SOCE-mediated Ca^2+^ signaling in NPC by activating MAPK/ERK signaling. We confirmed this in vitro by showing that EBV infection increased levels of p-ERK1/2, while STIM1 knockdown reduced them. EBV-encoded latent membrane protein 1 has been shown to activate the MAPK/ERK pathway in NPC cells and thereby increase VEGF secretion, ultimately stimulating tumor angiogenesis [26,33]. Similarly, the MAPK/ERK pathway has been shown to drive VEGF production and angiogenesis in colorectal cancer [34,35].

**Figure 7:**
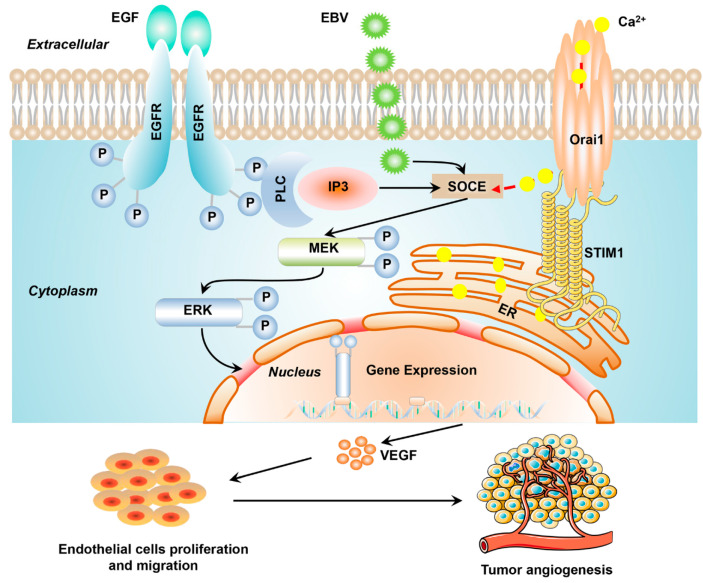
Schematic illustrating how STIM1-dependent Ca^2+^ signaling may mediate EBV-promoted tumor angiogenesis in NPC.

The present study helps clarify how EBV promotes angiogenesis by modulating STIM1-dependent Ca^2+^ signaling (Figure 7). Nevertheless, our results should be interpreted with caution given the following limitations. First, EBV-positive NPC cells in culture may not reflect all the properties of EBV infection in NPC patients. Second, we did not explore the potential involvement of Ca^2+^/calmodulin-dependent protein kinase II in the mechanisms stimulated by EBV here, even though the protein has been linked to the effect of Ca^2+^ signaling. Third, we did not verify the involvement of the ERK1/2 pathway in tumor angiogenesis by modulating the upstream/downstream proteins of ERK1/2 or inhibiting them chemically. Fourth, we did not examine the contribution of endothelial cells to EBV- and STIM1-driven processes in our NPC cells. Future studies should examine this, particularly the VEGFR activation of endothelial cells [25].

Despite these limitations, our study suggests that EBV infection enhances EGF-induced, SOCE-mediated Ca^2+^ signaling to activate ERK1/2 signaling and thereby promote angiogenesis in NPC. This activation depends on STIM1, since knocking down STIM1 decreases VEGF production and inhibits EBV-promoted angiogenesis. These findings may help guide further research into the pathophysiology of NPC and lead to novel treatments.

## Data Availability

Data are provided within the article.

## Supplementary Materials

The following are available online at https://www.mdpi.com/article/10.3390/pathogens10101275/s1, Figure S1: ErbB signaling pathway diagram, Figure S2: Representative results of immunohistochemistry against CD31 in each paraffin-embedded xenograft, Table S1: The basic characteristics of patients with NPC.
