# Supplementary material for: Epstein-Barr Virus Promotes Tumor Angiogenesis by Activating STIM1-Dependent Ca2+ Signaling in Nasopharyngeal Carcinoma
Source: Pathogens. 2021 Oct 3;10(10):1275. doi: 10.3390/pathogens10101275 (PMC8537240; doi:10.3390/pathogens10101275)
Supplement: Supplementary file 1 [file pathogens-10-01275-s001.zip › pathogens-1387965-supplementary.pdf]

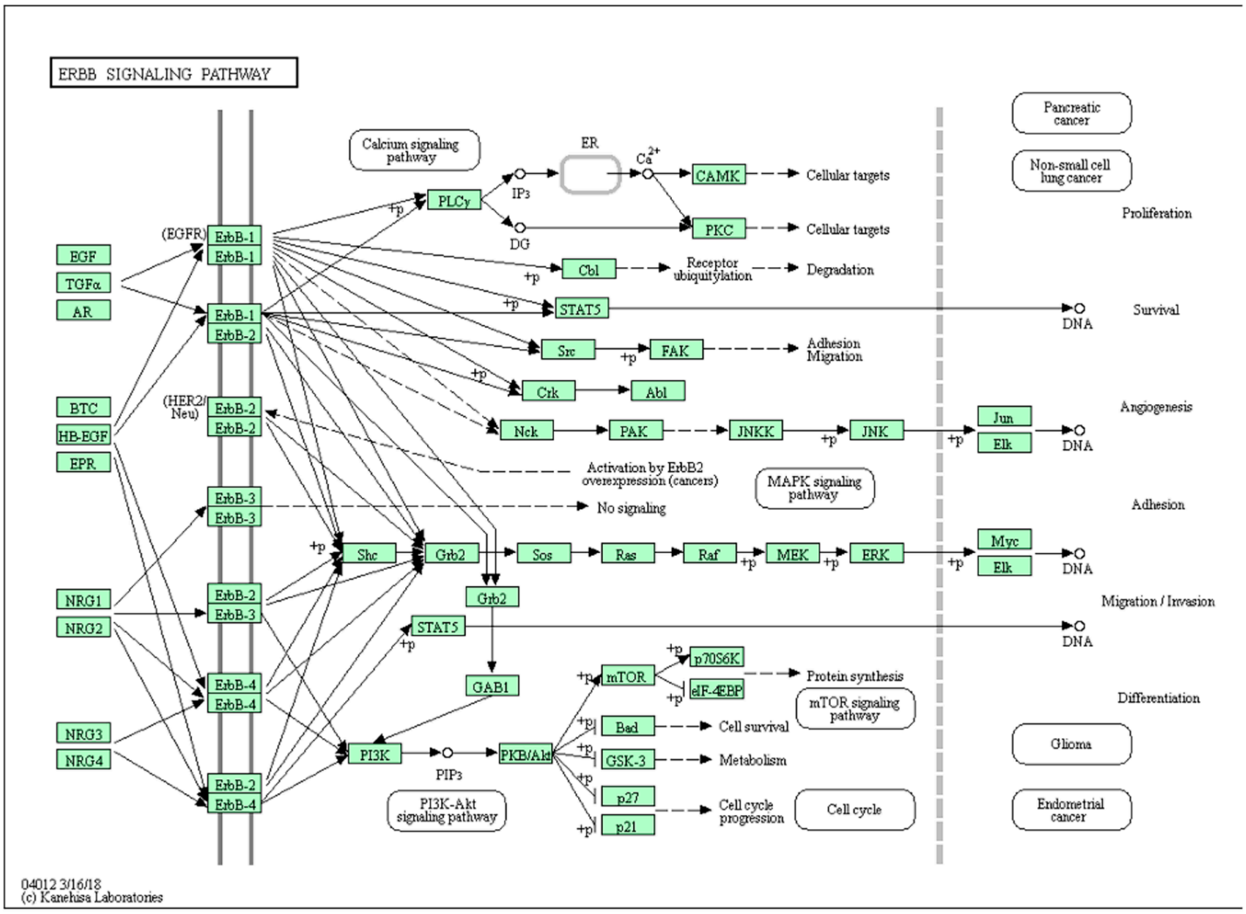

Figure S1. ErbB signaling pathway diagram.

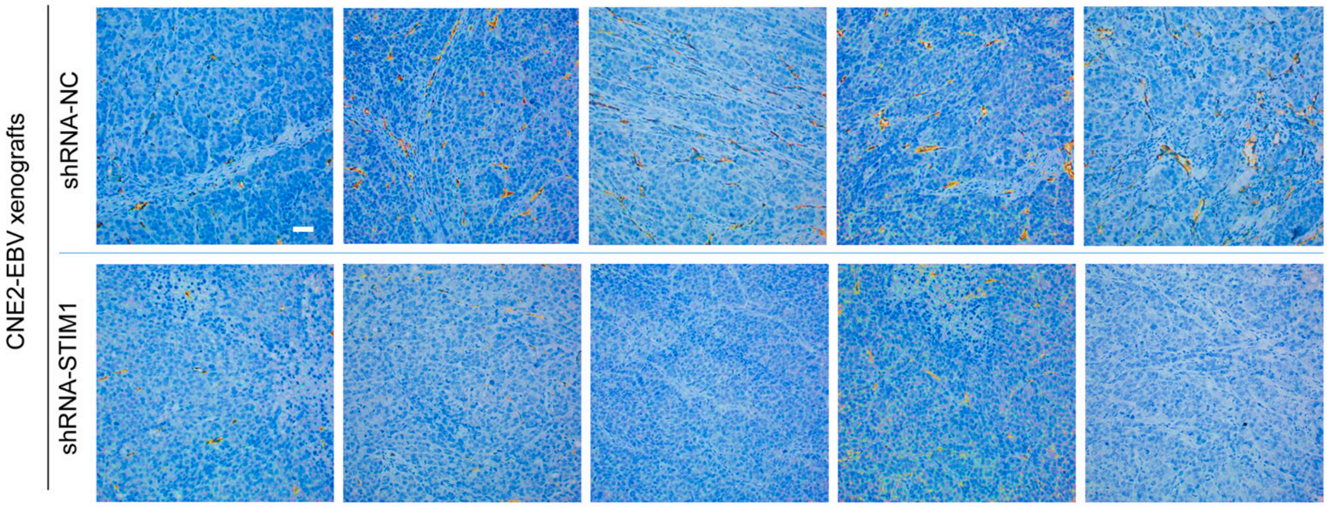

Figure S2. Representative results of immunohistochemistry against CD31 in each paraffin-embedded xenograft. The representative images correspond to all mice of Figure 3B from left to right. Scale bar = 100 μm.

Table S1. The basic characteristics of patients with NPC.

	EBV(−)	EBV(+)	*p*-Value
Gender			> 0.05
male	11	9	
female	3	6	
Age	50.7 ± 14.9	49.0 ± 13.6	> 0.05
Stage*			> 0.05
I-II	5	3	
III-IV	9	12	

Notes: NPC, nasopharyngeal carcinoma; * The Union for International Cancer Control (7th edition) staging system.
